# Predictive Models of Assistance Dog Training Outcomes Using the Canine Behavioral Assessment and Research Questionnaire and a Standardized Temperament Evaluation

**DOI:** 10.3389/fvets.2019.00049

**Published:** 2019-02-27

**Authors:** Emily E. Bray, Kerinne M. Levy, Brenda S. Kennedy, Deborah L. Duffy, James A. Serpell, Evan L. MacLean

**Affiliations:** ^1^Arizona Canine Cognition Center, School of Anthropology, University of Arizona, Tucson, AZ, United States; ^2^Canine Companions for Independence, National Headquarters, Santa Rosa, CA, United States; ^3^Office of Institutional Research and Effectiveness, University of the Arts, Philadelphia, PA, United States; ^4^Department of Clinical Sciences and Advanced Medicine, School of Veterinary Medicine, University of Pennsylvania, Philadelphia, PA, United States; ^5^Department of Psychology, University of Arizona, Tucson, AZ, United States

**Keywords:** C-BARQ, canine, assistance dogs, prediction, temperament, behavior, service animal

## Abstract

Assistance dogs can greatly improve the lives of people with disabilities. However, a large proportion of dogs bred and trained for this purpose are deemed unable to successfully fulfill the behavioral demands of this role. Often, this determination is not finalized until weeks or even months into training, when the dog is close to 2 years old. Thus, there is an urgent need to develop objective selection protocols that can identify dogs most and least likely to succeed, from early in the training process. We assessed the predictive validity of two candidate measures employed by Canine Companions for Independence (CCI), a national assistance dog organization headquartered in Santa Rosa, CA. For more than a decade, CCI has collected data on their population using the Canine Behavioral Assessment and Research Questionnaire (C-BARQ) and a standardized temperament assessment known internally as the In-For-Training (IFT) test, which is conducted at the beginning of professional training. Data from both measures were divided into independent training and test datasets, with the training data used for variable selection and cross-validation. We developed three predictive models in which we predicted success or release from the training program using C-BARQ scores (*N* = 3,569), IFT scores (*N* = 5,967), and a combination of scores from both instruments (*N* = 2,990). All three final models performed significantly better than the null expectation when applied to the test data, with overall accuracies ranging from 64 to 68%. Model predictions were most accurate for dogs predicted to have the lowest probability of success (ranging from 85 to 92% accurate for dogs in the lowest 10% of predicted probabilities), and moderately accurate for identifying the dogs most likely to succeed (ranging from 62 to 72% for dogs in the top 10% of predicted probabilities). Combining C-BARQ and IFT predictors into a single model did not improve overall accuracy, although it did improve accuracy for dogs in the lowest 20% of predicted probabilities. Our results suggest that both types of assessments have the potential to be used as powerful screening tools, thereby allowing more efficient allocation of resources in assistance dog selection and training.

## Introduction

Assistance dogs can greatly improve the lives of people with disabilities. By performing tasks such as picking up dropped items, opening doors, and turning on and off lights, they allow their handlers to approach life with greater independence and confidence. However, even among dogs that are specifically bred for these tasks, the rate of success typically ranges from 30 to 50% (1). At Canine Companions for Independence (CCI)—the largest nonprofit provider of assistance dogs for people with physical disabilities in the United States–the success rate over the past 13 years has averaged 43% when breeders and medical releases are excluded (K. Levy, personal communication, November 26, 2018). To be successful, these dogs must be robust to environmental stressors (large crowds, loud noises) and distractions (other animals and people, food on the ground), and exhibit impulse control, flexible and sustained attention, appropriate social behavior, and independent problem solving. Given the extensive resources required to raise and train these dogs, predicting the development and proficiency of these skills as early as possible is crucial to saving time and expense, while ensuring productive placements.

To this end, researchers have turned to a variety of tools in order to find early precursors of success: questionnaires that ask owners, raisers, or trainers to rate a dog's behavior [e.g., (2, 3)] and early environment [e.g., (4)], tracking of physiological measures (5), observations of maternal style (6, 7), batteries of temperament tests [e.g., (8, 9)], and measurements of cognitive variability through test batteries (10–12) and fMRI brain scans (13).

For the past 13 years, two formalized methods of evaluation that take no more than 15 min per dog have been regularly implemented in the dog population at CCI, an organization that breeds, trains, and places assistance dogs. The first is a standardized behavioral questionnaire that is completed by volunteer puppy raisers that care for each dog from 8 weeks of age until the dog returns for professional training (~18 months). The Canine Behavioral Assessment and Research Questionnaire (C-BARQ , www.cbarq.org), consisting of 100 items, was developed and validated for guide dogs (14) and pet dogs (15), and is now widely and systematically used among assistance dog organizations (1, 12). This method of assessment is advantageous in that it is easy to collect large amounts of data that provide a glimpse into each dog's behavioral profile prior to the dog entering training, with this information provided by the person who has been raising and observing the dog from 8 weeks of age. On the other hand, these measures include a degree of subjectivity, may not be available for all dogs (depending on puppy raiser compliance), can be noisy because every dog is evaluated by a different person, and it is impossible to confirm the accuracy of responses.

Secondly, CCI also conducts a standardized temperament test known as the In-For-Training (IFT) test, when dogs return to training campuses for professional training (16). The IFT is similar to behavioral tests that have previously been used by working dog groups in Sweden (17) and the UK (18). Like the C-BARQ, the IFT is characterized by distinct strengths and limitations. IFT scores are determined by a much smaller pool of trained evaluators who record behavior under experimental conditions using a clearly defined rubric. However, dog behavior and test results may be affected by uncontrolled variables, such as minor differences in the test procedure across time or location, variation in weather, or external distractions.

Past research has uncovered associations between questionnaire-reported assessments of behavior and working dog outcomes. Arata et al. (19) had trainers fill out questionnaires 3 months into training and found that the reported measure of distraction was especially effective at predicting guide dog outcome. Harvey et al. (20) developed and validated a questionnaire for guide dog trainers, then created a predictive model in which traits such as adaptability, body sensitivity, distractibility, excitability, general anxiety, trainability, and stair anxiety showed the potential to predict later outcomes. In another study spanning five working dog organizations (including CCI) that used the C-BARQ specifically, Duffy and Serpell (1) found significant associations between favorable raiser-reported scores and successful program outcome on 27 out of 36 traits. Thus, while many studies have described robust associations between aspects of behavior and temperament and training outcomes, few studies have developed and tested predictive models for forecasting these outcomes [but see (20)].

Additionally, researchers have found relationships between working dog success and temperament tests with similar components to the IFT. In a pilot study, Batt et al. (21) found that measures of reactivity at 14 months were associated with ultimate guide dog success. Harvey et al. (18) conducted a temperament test at 8 months of age and found that 5 of 11 behavioral measures were associated with success in a guide dog program, including posture when meeting a stranger, reaction to and chase behavior toward novel objects, and playfulness with a tea towel. Other researchers have found associations between temperament measures and later guide dog success as early as 8 weeks of age (22). However, to our knowledge, data from the specific IFT test implemented by CCI has never been used to predict whether a dog will graduate.

In the current work, we conducted a formal prediction study to determine how effectively we could predict which dogs would graduate as assistance dogs or be released from the program for behavioral reasons. As the predictor variables, we used C-BARQ scores collected by puppy raisers around 12 months of age (Experiment 1), behavioral IFT evaluations assessed by trainers around 18 months of age (Experiment 2), and a combination of both assessment types (Experiment 3).

## General Methods

### Subjects

All dogs in the study were Labrador retrievers, Golden retrievers, or crosses of the two breeds purpose-bred by CCI. CCI granted informed consent to all aspects of the study. CCI is a non-profit assistance dog organization that places service dogs (with adults with physical disabilities), skilled companions (with a team consisting of an adult or child with a disability and a facilitator), facility dogs (with a facilitator in a health care or educational setting), hearing dogs (with an adult who is deaf or hard of hearing), and service dogs for veterans (with physical disabilities or post-traumatic stress disorder). CCI has a nationwide presence; their national headquarters and Northwest Region Training Center are in Santa Rosa, CA (est. 1975) with additional training centers in Oceanside, CA (est. 1986), Delaware, OH (est. 1987), Orlando, FL (est. 1989), Medford, NY (est. 1989), and Irving, TX (est. 2016). Dogs in CCI's program are whelped in volunteer breeder-caretaker homes in Northern CA. Around 8 weeks of age, dogs are placed with volunteer puppy raisers across the country who care for dogs in their homes until the dogs are ~18 months of age, at which point they are sent to one of CCI's regional centers to begin professional training.

Participating dogs were born between the years of 2004 and 2017. To be eligible for the study, dogs needed to have a C-BARQ completed around 1 year of age by their puppy raiser (Experiment 1), participated in the In-For-Training behavioral test administered by CCI staff at their respective campus around 18 months of age (Experiment 2), or met both requirements (Experiment 3). Additionally, since we were interested in predicting behavioral suitability for assistance work, we only included dogs that succeeded in being placed for at least 1 year or were released from the program for behavioral reasons (e.g., distractibility, anxiety, fear, reactivity, sensitivity). Breeders were excluded from analysis, as were dogs released solely for medical reasons, consistent with previous studies on cognitive, behavioral, and temperamental predictors of working dog outcomes [e.g., (7, 10)]. Hearing dogs were excluded from analysis as they are selected for a different behavioral phenotype than the other roles (10), and they are only trained at a subset of the campuses and thus not representative of the population at a national level. Finally, dogs placed with veterans with post-traumatic stress disorder and dogs from the newest campus in Irving, TX were excluded from analysis due to insufficient sample size.

### Missing Data Imputation

For all instances where baseline values were missing, we used an imputation strategy based on a random forest [missForest package in R; (23)]. This method uses bootstrap aggregation of regression trees, which results in less biased parameters than parametric methods using linear regression, and also decreases the risk of overfitting (24). We imputed missing values using all baseline predictors, as well as outcome data and demographic variables accounting for sex, breed, coat color, training region, and the year that the dog entered training. When imputing missing baseline values, including outcomes ensures that the coefficients are closest to “true” coefficients, whereas excluding outcomes leads to biased (underestimated) coefficients (25). We imputed our “training” and “test” datasets separately.

### Statistical Analysis

Each dataset was divided into independent training and test data, using 2/3 of the data for variable selection and cross-validation, and 1/3 of the data for assessing predictive validity with an independent sample. As additional covariates we included sex, breed, coat color, training region, and year (in 2-year increments) that the dog entered training. We initially assessed a variety of modeling strategies with each of the different training datasets (Experiments 1–3) to determine what type of model might be most appropriate for these data. Specifically, we performed preliminary modeling using a generalized linear model, linear discriminant analysis, regularized regression (elastic net), partial least squares, and a k-nearest neighbors approach. Within the training data, the performance of these models was evaluated using 4-fold cross-validation repeated 10 times (data randomly divided into 4-folds, 3-folds used for model construction, 1-fold used to assess model accuracy, with this process repeated 10 times). As a measure of performance, we used the area under the curve (AUC) from the receiver operating characteristic, a measure of sensitivity and specificity for a binary classifier. AUC values range between 0.5 and 1, with a value of 0.5 indicating a non-informative model, and a value of 1 indicating a perfectly predictive model. Categorical predictions (graduate, release) were made using a probability threshold of 0.5 (i.e., predict release when predicted probability of graduation <0.5; predict graduate when predicted probability of graduation > 0.5.) Across the different training datasets, a general linear model performed as well or better than all other model types, and thus we used this approach for predictions with the test data. Variables were selected for the generalized linear model using a recursive feature elimination approach (with the training data), as implemented in the caret R package (26, 27).

For the test data, we predicted training outcomes using a model fit to all of the training data, and again used a probability threshold of 0.5 for predicting whether dogs in the test dataset would graduate from the program. In addition to these categorical predictions, we retained the predicted probabilities of graduation for each dog in the test dataset in order to explore accuracy across the range of predicted probabilities. These predicted probabilities were divided into deciles (i.e., 1st decile corresponding to the 10% of the test sample predicted to have the lowest probability of success, 10th decile corresponding to the 10% of the test sample predicted to have the highest probability of success). We then assessed accuracy across deciles to identify probability regions where the predictive model was most and least accurate. To identify which terms made the most important contributions to the model, we assessed a measure of variable importance, defined as the absolute value of the z-statistic for each term in the model (27). Overall model performance was measured using accuracy and the AUC from the receiver operating characteristic. To test whether model predictions were better than the null expectation, we performed a one-tailed binomial test to assess whether accuracy was significantly higher than the “no information rate” (the accuracy which could be obtained by predicting the majority class for all observations).

## Experiment 1

### Methods

#### Subjects

A request to fill out a C-BARQ questionnaire was sent to puppy raisers via email by CCI when the dog turned 1 year of age. Completion of the questionnaire implied informed consent. Most puppy raisers completed an online version of the survey through the website (www.cbarq.com), although they were also given the option to fill out the same survey on paper and return via mail. These surveys take approximately 10–15 min to complete and were filled out while the dog was still living with the puppy raiser, prior to being returned to campus for professional training. Dogs whose questionnaires were completed after their 2nd birthday (*N* = 17) and dogs missing data on more than 4 variables (*N* = 74) were excluded from analysis. In total, there were 3,569 dogs that met our criteria with a completed C-BARQ questionnaire and a behavioral outcome (1,715 females, 1,854 males; 707 Labrador retrievers, 193 Golden retrievers, 2,669 Labrador × Golden crosses). The average age at evaluation was 58.3 ± 8.4 weeks. In our sample, 60% of subjects were behavioral releases (*N* = 2,132).

#### Measures

The C-BARQ is particularly focused on assessing the frequency and severity of problematic behaviors (28). It consists of several miscellaneous items as well as 14 different categories of behavior—stranger-directed aggression, owner-directed aggression, dog-directed aggression, stranger-directed fear, non-social fear, dog-directed fear, separation-related behavior, attachment and attention-seeking, trainability, chasing, excitability, touch sensitivity, energy level, and dog rivalry—originally extracted by factor analysis (1, 15). Scores on these categories are obtained by averaging scores across raw test items assessing behaviors relevant to these constructs (see Appendix A). Dogs only received a score in a given category if at least 80% or greater of the scores that made up the category were recorded (1).

Among the 3,569 questionnaires analyzed in the current study, we only included items that were recorded for 90% or more of participants. Using this cut-off criteria, we dropped the following measures from analysis: chasing other animals (miscellaneous items 74–76), escape behavior (miscellaneous item 77), and rolling in smelly substances (miscellaneous item 78).

#### Analysis

Data preparation and analysis followed the procedure described in sections Missing Data Imputation and Statistical Analysis.

### Results and Discussion

Initial modeling using the training dataset and C-BARQ measures as predictor variables yielded a cross-validated accuracy of 0.65. Estimates, standard errors, z-values, and *p-*values of the C-BARQ predictors are presented in Table 1. The five C-BARQ variables of most importance to the final model (in order of importance) included: barking (lower levels predicted higher probability of graduation), stranger-directed fear (lower levels predicted higher probability of graduation), dog-directed aggression (lower levels predicted higher probability of graduation), coprophagia (higher levels predicted higher probability of graduation), and trainability (higher levels predicted higher probability of graduation). Fitting this model to the test data, outcomes were predicted with an overall accuracy of 0.68, yielding an AUC of 0.71. Overall, model predictions were significantly better than the null expectation (no information rate = 0.60; *p* < 0.01).

**Table 1:** Estimates, standard errors, z-values, and *p* values from the GLM used in Experiment 1 in which the dependent variable was outcome in the assistance dog program and CBARQ scores were the predictor variables.

**Predictor variables (C-BARQ scores)**	**Estimate**	**Std. error**	**z value**	**Pr(>|z|)**
Intercept	1.84	0.30	6.03	0.00
Barks persistently when alarmed or excited	0.23	0.06	3.71	0.00
Stranger-directed fear	0.28	0.08	3.64	0.00
Dog-directed aggression	0.26	0.07	3.61	0.00
Coprophagia	−0.16	0.05	−3.26	0.00
Trainability	−0.16	0.05	−2.88	0.00
Pulls on leash	0.16	0.06	2.87	0.00
Begs persistently for food	−0.13	0.05	−2.37	0.02
Chews inappropriate objects	0.12	0.05	2.30	0.02
Fear of stairs	0.12	0.05	2.29	0.02
Separation-related behavior	0.13	0.06	2.23	0.03
Urinates when approached, petted, or handled	0.11	0.06	2.00	0.05
Energy level	0.11	0.06	1.88	0.06
Licks him/herself excessively	−0.10	0.06	−1.82	0.07
Stares intently at nothing visible	−0.09	0.05	−1.74	0.08
Displays bizarre, strange, or repetitive behaviors	0.09	0.06	1.67	0.10
Dog rivalry	−0.11	0.07	−1.60	0.11
Steals food	0.09	0.06	1.56	0.12
Touch sensitivity	−0.08	0.05	−1.51	0.13
Attachment and attention-seeking behaviors	−0.08	0.05	−1.50	0.13
Defecates when left alone	0.08	0.05	1.47	0.14
Owner-directed aggression	0.12	0.08	1.46	0.15
Hyperactive	0.08	0.06	1.36	0.17
Snaps at (invisible) flies	−0.07	0.05	−1.32	0.19
Mounts objects, furniture, or people	−0.06	0.05	−1.15	0.25
Excitability	0.06	0.06	1.07	0.28
Dog-directed fear	−0.04	0.06	−0.65	0.51
Tail-chasing	−0.03	0.05	−0.55	0.58
Non-social fear	0.03	0.06	0.48	0.63
Urinates when left alone	0.02	0.05	0.41	0.68
Licks people or objects excessively	0.02	0.05	0.36	0.72
Stranger-directed aggression	−0.02	0.07	−0.21	0.84

**Figure 1:**
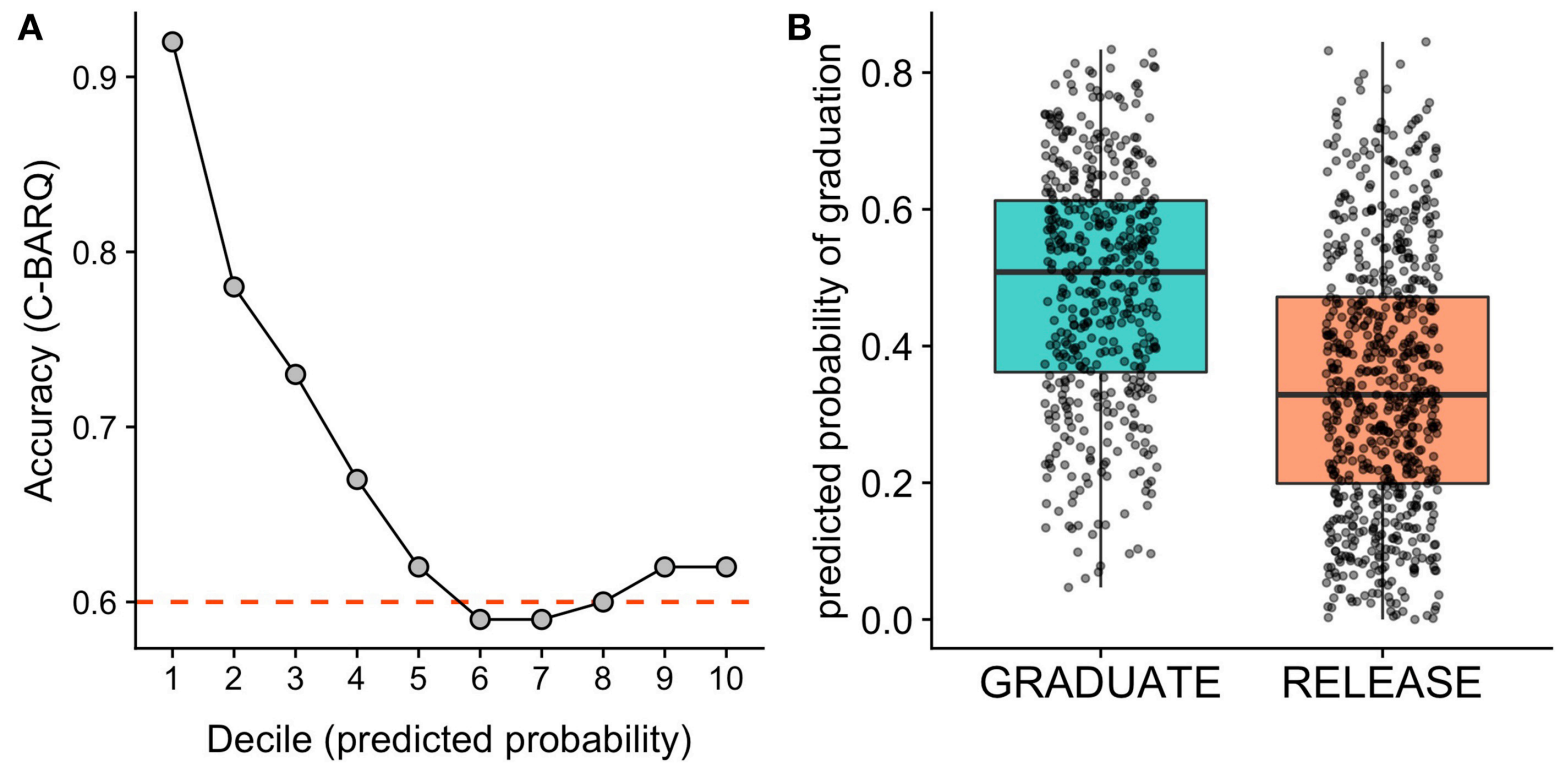
Results of models using the C-BARQ to predict assistance dog training outcomes. **(A)** Model accuracy as a function of deciles of the predicted probability of graduation for the test sample. The model was most accurate at identifying dogs with the lowest probability of success. The red dashed line indicates the No Information Rate (NIR), the accuracy that could be obtained by predicting the majority class for all observations. The C-BARQ predictive model performed significantly better than the NIR. **(B)** Predicted probabilities of graduation for dogs that ultimately graduated or were released from the program. Points overlaid on the boxplots reflect predicted probabilities for individual dogs. Horizonal jittering of points and transparency are used to reduce overplotting.

Assessing accuracy across deciles of the predicted probability of success, we found that the dogs least likely to succeed in training could be identified with a remarkably high accuracy. Specifically, for the 10% of dogs predicted to be least likely to succeed, model predictions were 92% accurate. For dogs in the lowest 20% of predicted probabilities, accuracy was 85% (Figure 1). In contrast, for the dogs predicted to have the highest probability of success, predictions were much less accurate (62% accuracy for dogs in the top decile of predicted probabilities). This pattern of results is consistent with the intended purpose of the C-BARQ, which was designed primarily to identify problematic behaviors (15, 29). Thus, from an applied perspective, the C-BARQ may be most useful for identifying the dogs that are least likely to succeed. Given that dogs with the lowest probability of success can be identified with a high accuracy, the C-BARQ has potential to be a powerful screening tool that can be incorporated prior to the commencement of formal training.

## Experiment 2

### Methods

#### Subjects

Subjects included dogs that had completed an In-For-Training Evaluation (IFT) around 18 months of age. As in Experiment 1, dogs missing data for more than 4 variables (*N* = 61) were excluded from analysis. In total, there were 5,967 dogs that met our criteria with IFT test participation and a behavioral outcome (2,892 females, 3,075 males; 1,249 Labrador retrievers, 265 Golden retrievers, 4,453 Labrador × Golden crosses). The mean age at evaluation was 1.6 ± 0.1 years. In our sample, 58% of subjects were behavioral releases (*N* = 3,489).

#### Measures

The IFT test occurs on a single morning the week after dogs arrive at campus to begin professional training and takes ~10 min per dog. In the IFT test, the dog is exposed to six scenarios: a physical exam, a looming object, a sudden noise, a ‘prey’ object, an unfamiliar dog, and a threatening stranger. These scenarios were chosen to be stimulating enough to potentially elicit problematic behaviors, while remaining within the realm of normal occurrences that a dog might conceivably face in his/her working life. In the physical exam portion, the dog is handled by a stranger as if at a veterinary examination, culminating in the tester attempting to roll the dog over onto his/her side without any commands being given. In the looming object portion, a trash bag unexpectedly falls toward the dog from a height of 3–4 feet. In the sudden noise portion, a heavy chain is dragged across metal for ~2–3 s. In the “prey” object portion, a rag on a string is erratically moved away from the dog, who is given the opportunity to chase it. In the unfamiliar dog portion, the dog is led toward a life-sized stuffed Old English sheepdog (30). In the threatening stranger portion, the dog is led toward a hooded figure who is hunched over, striking a cane against the ground, and yelling (30). In each of these scenarios, the dog's reaction, recovery (where applicable), and body language is coded (see Appendix B). Across scenarios, low scores correspond to appropriate behavior, while higher scores indicate visible discomfort, reactivity, and failure to recover.

Among the 5,967 IFT tests included in the current study, scores on all items were recorded for 95% or more of participants. The only measure that was dropped from analysis was the categorization of the dog's general demeanor during the physical exam portion, since it was the only categorical variable.

#### Analysis

Data preparation and analysis followed the procedure described in sections Missing Data Imputation and Statistical Analysis.

### Results and Discussion

Initial modeling using the training dataset and IFT measures as predictor variables yielded a cross-validated accuracy of 0.64. Estimates, standard errors, z-values, and *p* values of the IFT predictors are presented in Table 2. The five IFT variables of most importance to the final model (in order of importance) included: body tension during the physical exam (lower scores—i.e., more relaxed—predicted higher probability of graduation), behavior during the second pass following the sudden noise (referred to as “conclusion” phase in Appendix B; lower scores—i.e., less reactivity—predicted higher probability of graduation), recall after confronting the unfamiliar dog (lower scores—i.e., readily leaves—predicted higher probability of graduation), initial reaction during the prey test (lower scores—i.e., less reactivity—predicted higher probability of graduation), and response to handling during the physical exam (lower scores—i.e., lower resistance—predicted higher probability of graduation). Fitting this model to the test data, outcomes were predicted with an overall accuracy of 0.66, yielding an AUC of 0.71. Overall, model predictions were significantly better than chance expectation (no information rate = 0.58; *p* < 0.01).

**Table 2:** Estimates, standard errors, z-values, and *p* values from the GLM used in Experiment 2 in which the dependent variable was outcome in the assistance dog program and IFT scores were the predictor variables.

**Predictor variables (IFT scores)**	**Estimate**	**Std. error**	**z value**	**Pr(>|z|)**
Intercept	2.89	0.47	6.09	0.00
Physical exam: body tension	0.16	0.04	3.87	0.00
Sudden noise: conclusion	0.15	0.05	3.32	0.00
Unfamiliar dog: recall	0.12	0.04	2.80	0.01
Prey: initial reaction	0.17	0.07	2.51	0.01
Sudden noise: initial reaction	0.11	0.04	2.47	0.01
Physical exam: ease of handling	0.10	0.04	2.36	0.02
Unfamiliar dog: initial reaction	0.09	0.04	2.21	0.03
Looming object: initial reaction	0.09	0.04	2.14	0.03
Unfamiliar dog: tail position	0.16	0.08	1.90	0.06
Looming object: second walk by	0.25	0.15	1.65	0.10
Sudden noise: barks or growls	1.15	0.77	1.49	0.14
Looming object: increase in activity	0.25	0.17	1.48	0.14
Threatening stranger: initial reaction	0.07	0.05	1.46	0.14
Threatening stranger: recovery	0.06	0.04	1.43	0.15
Prey: conclusion	0.06	0.05	1.14	0.26
Threatening stranger: increase in activity	0.08	0.07	1.12	0.26
Threatening stranger: barks or growls	0.13	0.14	0.95	0.34
Physical exam: vocalization	0.03	0.04	0.87	0.38
Unfamiliar dog: barks or growls	0.14	0.17	0.85	0.40
Prey: recovery	−0.04	0.08	−0.51	0.61

**Figure 2:**
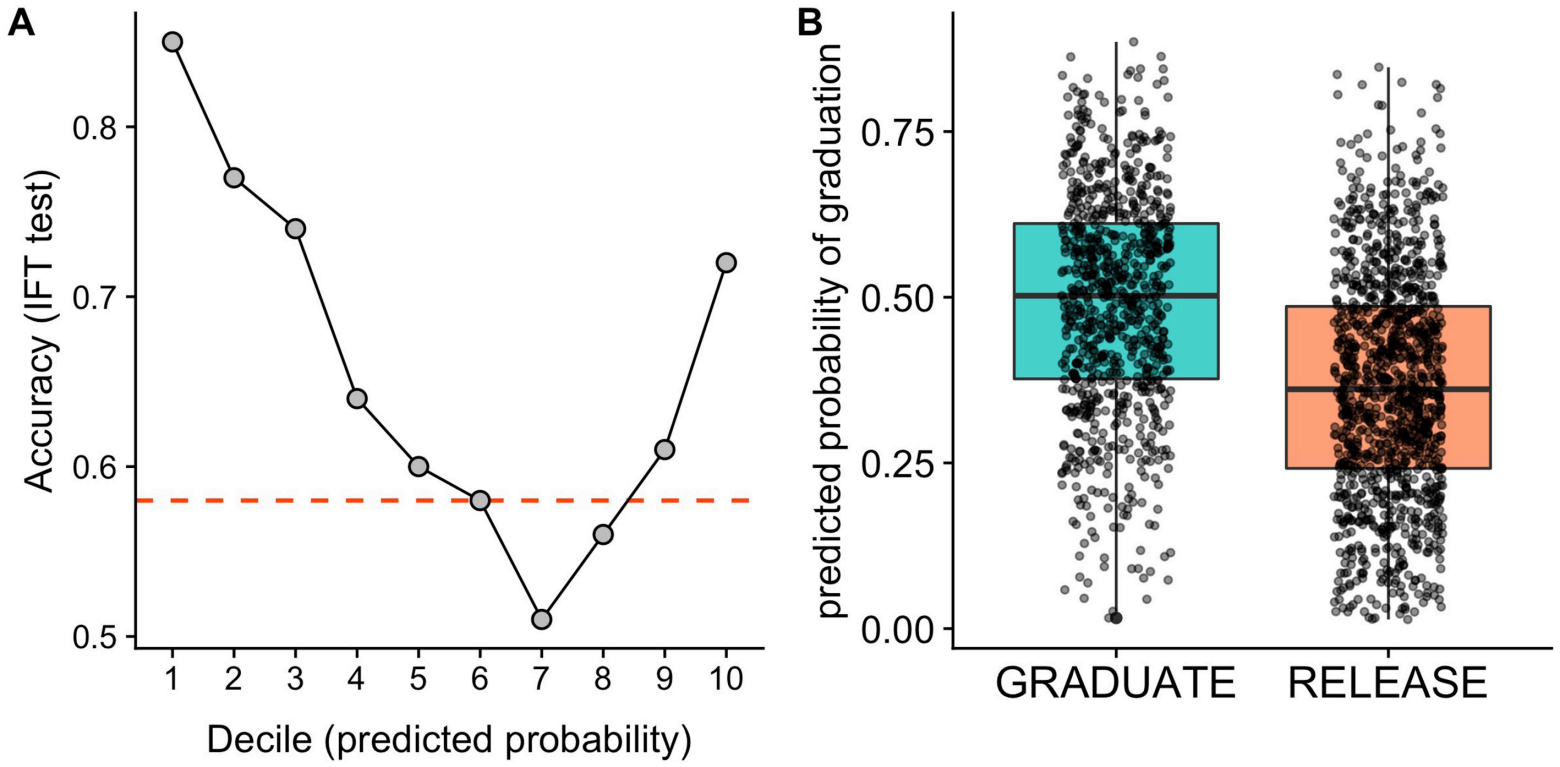
Results of models using the In-For-Training (IFT) temperament test to predict assistance dog training outcomes. **(A)** Model accuracy as a function of deciles of the predicted probability of graduation for the test sample. The model was most accurate at identifying dogs with the lowest probability of success, but also useful for identifying dogs with the highest probability of success. The red dashed line indicates the No Information Rate (NIR), the accuracy that could be obtained by predicting the majority class for all observations. The IFT predictive model performed significantly better than the NIR. **(B)** Predicted probabilities of graduation for dogs that ultimately graduated or were released from the program. Points overlaid on the boxplots reflect predicted probabilities for individual dogs. Horizonal jittering of points and transparency are used to reduce overplotting.

Assessing accuracy across deciles of the predicted probability of success, we found that the dogs least likely to succeed in training could be identified with a high accuracy based on IFT measures. For the 10% of dogs predicted to be least likely to succeed, model predictions were 85% accurate, and for dogs in the lowest 20% of predicted probabilities, accuracy was 81% (Figure 2). Accuracy using the IFT model was also reasonably high for the group of dogs predicted to have the highest probability of success. For the 10% of dogs predicted to be most likely to succeed, prediction accuracy was 72%. Therefore, while the most accurate predictions from the IFT concerned the dogs least likely to succeed, these data were also useful for identifying an elite group of dogs most likely to graduate from the program. Because the IFT is completed after dogs have returned to the training center, but before a large investment in professional training, our findings suggest that outcome predictions based on the IFT may help to streamline and expedite decisions about which dogs to retain for subsequent professional training or breeding purposes.

## Experiment 3

Because Experiments 1–2 suggested that the C-BARQ and IFT were both useful measures for predicting training outcomes, in Experiment 3 we investigated whether predictive accuracy could be improved by combining data from both instruments. Because not all dogs had data for both the C-BARQ and IFT, these analyses were restricted to a slightly smaller subset of dogs for which both measures were available.

### Methods

#### Subjects

Participants in Experiment 3 consisted of the dogs from Experiments 1–2 who had 12-month C-BARQ scores, 18-month IFT test scores, and a behavioral outcome. In total, there were 2,990 dogs that met these criteria (1,453 females, 1,537 males; 599 Labrador retrievers, 149 Golden retrievers, 2,242 Labrador × Golden crosses). The mean age at evaluation for the CBARQ was 57.7 ± 8.0 weeks, and the mean age at evaluation for the IFT was 1.6 ± 0.1 years. In our sample, 59% of subjects were behavioral releases (*N* = 1,774).

#### Analysis

Because the sample in Experiment 3 differed from Experiments 1–2, we repeated analyses using the C-BARQ and IFT in isolation to obtain a baseline measure of accuracy using these measures in the sample for Experiment 3. We then performed analyses combining information from the C-BARQ and IFT to assess whether higher accuracy could be attained by leveraging both sets of predictor variables. These analyses were conducted in two ways. First, we developed a model using all variables from the C-BARQ and IFT as predictors. This approach exposed the model to all raw underlying variables simultaneously. Second, we fit separate models using the C-BARQ and IFT and saved predicted probabilities for each dog from these models. We then fit a final model using the predicted probabilities from the C-BARQ and IFT models as the predictor variables. Although this approach may be suboptimal from a statistical perspective (because not all variables are considered within the same model), it has the practical advantage that if one of the two data sources is missing, it remains possible to generate a predicted probability based on one of the two sets of predictor variables. In addition, because the final model has only two predictor variables (probability from the C-BARQ model, and probability from the IFT model), it is possible to assess which data source carries the most weight by inspecting the beta coefficients associated with each of these predictors.

### Results and Discussion

**Figure 3:**
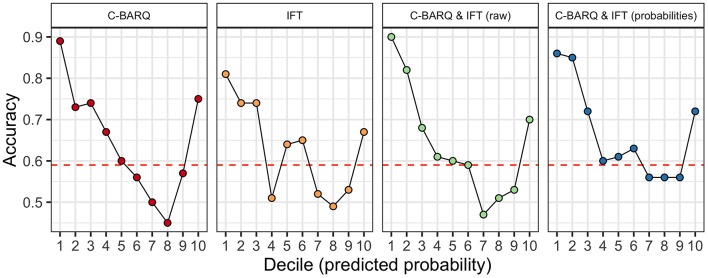
Results of models for a subset of the data (*N* = 2,990) for which both C-BARQ and In For Training (IFT) scores were available. All panels depict accuracy as a function of deciles of the predicted probability of graduation for the test sample. The red dashed line indicates the No Information Rate (NIR), the accuracy that could be obtained by predicting the majority class for all observations. The panels for C-BARQ and IFT show accuracy for this subset of dogs using the C-BARQ or IFT in isolation. The C-BARQ & IFT (raw) panel shows results from a model combining raw data from both measures. The C-BARQ & IFT (probabilities) panel shows results from a model using predicted probabilities from the stand-alone C-BARQ and IFT models as the predictor variables (see text for details).

Accuracy for the four models used in Experiment 3 is shown in Figure 3. The model using only the C-BARQ data had an accuracy of 0.65, and an AUC of 0.7, performing slightly worse than we observed using a larger sample in Experiment 1. The model using only the IFT data had an accuracy of 0.63 and an AUC of 0.65, again performing slightly worse than the IFT model fit to a larger dataset in Experiment 2. The model combining all C-BARQ and IFT predictors yielded an overall accuracy of 0.64, and an AUC of 0.69. Therefore, the combination of C-BARQ and IFT data actually led to poorer overall performance with this sample, than use of the C-BARQ alone. Lastly, the model using predicted probabilities from the stand-alone C-BARQ and IFT models yielded an accuracy of 0.67, and an AUC of 0.7. Thus, at least in this instance, there was no meaningful information loss in the model using separate probabilities from the IFT and C-BARQ as predictor variables, and in fact, this model outperformed all others.

As with the models from Experiments 1–2, accuracy varied as a function of the predicted probability of success for all models used in Experiment 3 (Figure 3). Specifically, all models were best at identifying dogs that were least likely to complete training and were moderately successful at predicting a smaller fraction of dogs that were most likely to complete training. For the dogs predicted to be in the 20% of the sample least likely to succeed (deciles 1 and 2), both models combining information from the C-BARQ and IFT outperformed models using the C-BARQ or IFT in isolation (accuracy collapsing across deciles 1–2: C-BARQ & IFT [raw data]: 86%; C-BARQ & IFT [probabilities]: 86%; C-BARQ alone: 81%; IFT alone: 78%). Therefore, while overall accuracy was not much higher when combining the C-BARQ and IFT, accuracy was appreciably higher with respect to identifying the dogs least likely to succeed. These findings suggest that leveraging both data sources provides an improved strategy for identifying these dogs, and that there is little difference between approaches including all predictors together in a single model vs. aggregating predicted probabilities from independent data sources.

To assess the relative importance of predictor variables from the C-BARQ and IFT, we determined variable importance from the model including raw data from both sets of measures and compared the beta coefficients from the model using predicted probabilities from each data source. Estimates, standard errors, z-values, and *p* values from the former model are presented in Table 3. The five most important variables included 3 C-BARQ measures (dog-directed aggression, barking, and chewing, where lower levels predicted higher probability of graduation) and two IFT measures (behavior during the second pass following the sudden noise and initial reaction to the looming object, where less reactivity predicted higher probability of graduation), suggesting that both data sources made important contributions to the model. For the model using independent probabilities based on the C-BARQ and IFT, the coefficients associated with each data source were comparable (C-BARQ: β = −3.30, IFT: β = −3.17) again suggesting that both sets of measures were important.

**Table 3:** Estimates, standard errors, z-values, and *p* values from the GLM used in Experiment 3 in which the dependent variable was outcome in the assistance dog program and CBARQ and IFT scores were the predictor variables.

**Predictor variables (CBARQ and IFT scores)**	**Estimate**	**Std. error**	**z value**	**Pr(>|z|)**
Intercept	17.00	1455.40	0.01	0.99
Dog-directed aggression	0.29	0.08	3.49	0.00
Barks persistently when alarmed or excited	0.21	0.07	3.08	0.00
Sudden Noise: conclusion	0.21	0.07	2.95	0.00
Chews inappropriate objects	0.18	0.06	2.94	0.00
Looming object: initial reaction	0.19	0.07	2.71	0.01
Stranger-directed fear	0.20	0.08	2.43	0.02
Begs persistently for food	−0.14	0.06	−2.27	0.02
Looming object: barks or growls	−1.29	0.58	−2.22	0.03
Threatening stranger: hackles	−0.58	0.26	−2.19	0.03
Unfamiliar dog: recall	0.14	0.06	2.17	0.03
Steals food	0.14	0.07	2.16	0.03
Prey: initial reaction	0.21	0.10	2.02	0.04
Physical Exam: body tension	0.16	0.08	1.95	0.05
Threatening stranger: initial reaction	0.14	0.07	1.92	0.06
Separation-related behavior	0.12	0.06	1.88	0.06
Fear of stairs	0.11	0.06	1.86	0.06
Prey: conclusion	0.15	0.08	1.82	0.07
Urinates when left alone	0.15	0.09	1.77	0.08
Coprophagia	−0.09	0.05	−1.68	0.09
Threatening stranger: recovery	0.11	0.07	1.57	0.12
Looming object: second walk by	0.39	0.25	1.54	0.12
Looming object: increase in activity	0.40	0.27	1.47	0.14
Displays bizarre, strange, or repetitive behaviors	0.09	0.06	1.44	0.15
Excitability	0.09	0.07	1.38	0.17
Touch sensitivity	−0.08	0.06	−1.37	0.17
Physical exam: ease of handling	0.09	0.07	1.37	0.17
Hyperactive	0.09	0.07	1.34	0.18
Trainability	−0.08	0.06	−1.28	0.20
Threatening stranger: increase in activity	−0.14	0.12	−1.18	0.24
Urinates against objects/furnishings in home	−0.07	0.06	−1.14	0.25
Snaps at (invisible) flies	−0.07	0.06	−1.10	0.27
Dog rivalry	−0.07	0.07	−0.97	0.33
Unfamiliar dog: barks or growls	0.27	0.28	0.96	0.34
Mounts objects, furniture, or people	−0.05	0.06	−0.91	0.36
Physical exam: vocalization	0.05	0.06	0.87	0.38
Dog-directed fear	−0.05	0.06	−0.79	0.43
Pulls on leash	0.05	0.06	0.77	0.44
Defecates when left alone	0.05	0.07	0.75	0.45
Unfamiliar dog: hackles	−0.15	0.20	−0.74	0.46
Stares intently at nothing visible	−0.04	0.06	−0.73	0.47
Stranger-directed aggression	0.06	0.09	0.70	0.48
Prey: recovery	−0.08	0.12	−0.67	0.51
Sudden noise: barks or growls	0.49	0.75	0.64	0.52
Unfamiliar dog: tail position	0.08	0.13	0.64	0.53
Owner-directed aggression	0.06	0.09	0.61	0.54
Licks people or objects excessively	0.04	0.06	0.60	0.55
Non-social fear	−0.03	0.06	−0.54	0.59
Threatening stranger: barks or growls	0.12	0.24	0.51	0.61
Attachment and attention-seeking behaviors	−0.03	0.06	−0.50	0.61
Energy level	0.03	0.07	0.48	0.63
Sudden noise: initial reaction	−0.03	0.07	−0.47	0.64
Looming object: recovery	0.03	0.07	0.42	0.68
Urinates when left alone	−0.02	0.06	−0.31	0.76
Tail-chasing	0.02	0.06	0.28	0.78
Licks him/herself excessively	−0.01	0.06	−0.19	0.85
Physical exam: tail position	0.01	0.07	0.16	0.87
Chases shadows	−0.01	0.06	−0.13	0.90
Sudden noise: recovery	0.01	0.07	0.11	0.91
Prey: barks or growls	0.08	1.35	0.06	0.95
Unfamiliar dog: initial reaction	0.00	0.06	0.05	0.96

## General Discussion

Although several previous studies have identified associations between behavioral or temperamental variables and working dog outcomes, few studies have moved beyond association to formal prediction of outcomes with an independent sample. For applied use, accurate prediction with novel cases provides the most important benchmark, because it addresses the accuracy with which a set of measures can forecast new events, rather than simply describing the past. For assistance dog providers, accurate predictive models can be used to guide decisions about which dogs to invest in, and which dogs are less likely to succeed. Using data from the C-BARQ and an internal temperament test (IFT), we found that statistical models using these instruments were useful for predicting training outcomes in an independent sample.

Notably, our models were best at identifying the dogs least likely to succeed and were less accurate at identifying dogs most likely to succeed. This finding is consistent with the design of the C-BARQ and IFT, which are intended to almost exclusively capture potentially problematic behaviors (e.g., barking, aggression, fear responses to novel stimuli). In contrast, recent studies using cognitive measures were best able to identify the dogs most likely to succeed, with less success at identifying dogs that would be released (10). Thus, a combination of data from diverse kinds of measures may prove most useful for identifying dogs that are both very likely, or very unlikely to succeed. The utility of combining different data sources is suggested by our findings in Experiment 3. Although overall predictions were not more accurate when combining information from the C-BARQ and IFT, the ability to identify dogs least likely to succeed improved considerably when incorporating both instruments. Therefore, an important challenge for future research will be to develop and integrate complementary measures, that together enhance predictive validity.

At a practical level, both of the measures we investigated can be obtained at minimal cost and collected rapidly across large samples of dogs. Specifically, data for the C-BARQ are provided by volunteer puppy raisers, placing no additional burden on professional dog trainers. This measure provides important information about a dog's behavioral profile, even before the dog arrives for professional training. Given that the C-BARQ was highly accurate at identifying the dogs least likely to succeed (92% accuracy for dogs in the lowest decile of probability of success), dog providers could potentially benefit by shifting focus away from these dogs prior to the commencement of professional training. In contrast to the C-BARQ, the IFT requires that a dog has returned to a professional training center and relies on evaluation by a professional dog trainer. Despite this modest increase in demands, the test itself is rapid, relies on observation under experimental conditions, and information is collected within 1 week of the dog's arrival for professional training. Given that the IFT was also highly accurate with respect to dogs least likely to succeed (85% accuracy for the lowest decile of probability of success), this measure provides another early opportunity for identifying which dogs warrant further investment.

Across experiments, our predictive models achieved high accuracy with respect to dogs least likely to succeed in training. However, the ultimate decision about what constitutes acceptable accuracy remains with dog providers, who must weigh the tradeoffs between correctly classifying a majority of cases, but at the cost of misclassifying the remaining minority. For example, using the model from Experiment 1, if 100 dogs in the lowest decile of probability of success were released prior to professional training, this would preempt investment in 92 of these dogs that ultimately would not succeed, but would also come at the cost of releasing 8 dogs that could have been successfully placed. To determine if such a tradeoff is worthwhile, organizations would need to consider the resources that could be devoted to breeding and raising additional dogs in lieu of those released based on a low probability of success. The financial and time costs of these decisions may vary widely across dog training organizations, and it is unlikely that there will be a one-size-fits-all solution.

Although we have emphasized the use of predictive models for the purposes of candidate assistance dog selection, another application for our findings relates to identifying phenotypic targets for selective breeding. A fundamental question in this area concerns the extent to which the traits that are predictive of outcomes are also heritable. If these traits exhibit substantial heritability, dog providers may consider these traits in breeder selection, with ultimate hopes of increasing the prevalence of favorable traits within the entire population of candidate dogs. Along these lines, several studies indicate that traits measured by the C-BARQ are moderately to strongly heritable (31–33), and traits similar to those measured in the IFT have been shown to be heritable in other populations (34, 35), suggesting promise for future developments in this area.

One important limitation of this work is that models were developed and applied within a single working dog population, and thus we cannot assess how well these results would generalize to other assistance dog agencies. This issue is especially important if other organizations breed, train, and evaluate dogs based on different target phenotypes. Indeed, previous studies investigating cognitive predictors of success as an assistance or explosive detection dog revealed a different set of traits predictive of outcomes in each population (10). Previous studies assessing associations between C-BARQ scores and outcomes in five large assistance dog associations revealed largely similar findings across dog providers, suggesting a common C-BARQ profile associated with assistance dog success (1). Nonetheless, future work will be required to develop and test predictive models for different organizations/training programs. Key questions in this area will consider the accuracy of prediction across organizations, as well as similarities and differences in which C-BARQ items are most useful for forecasting outcomes.

Among the specific C-BARQ findings from our study population, the puppy raiser's assessment of the dog's propensity to bark persistently when alarmed or excited was strongly predictive of later training outcomes; Dogs that exhibited this behavior more frequently were more likely to be released from the program. This finding corroborates recent results in guide dogs. Bray et al. (7) found that dogs who were quicker to vocalize in the presence of a novel, motion-activated stuffed cat (i.e., an occurrence that was likely perceived as exciting and/or alarming) were more likely to be released from the program, and similarly Harvey et al. (18) found that dogs least likely to graduate had higher scores on a principal component that accounted for time spent barking during the testing session. Taken together, these findings suggest that a tendency to be vocal is disadvantageous in assistance dogs—perhaps because vocalization is a useful proxy for some underlying trait, such as reactivity or anxiety, or because practically, it is an inappropriate behavior for a service animal. However, not all findings from our study were as intuitively interpretable. Perhaps most notably, higher levels of coprophagia (eating own or other animals' feces) were associated with higher odds of success as an assistance dog, despite the fact that coprophagic behavior is typically deemed undesirable and problematic for assistance dogs.

In sum, the current study suggests that assistance dog outcomes can be usefully predicted using measures from the C-BARQ and IFT, and that these predictions can be obtained prior to investment in formal professional training. These findings provide proof of concept for how assistance dog providers could use systematic data collection and predictive modeling to streamline the processes through which dogs are selected and bred for assistance work. In turn, improvements in these areas could reduce the substantial costs of assistance dog breeding and training, thereby increasing public health through more successful dog placement for people with disabilities and shorter waiting lists to receive these valuable placements.

## Ethics Statement

This study was carried out in accordance with the recommendations of the University of Arizona IACUC, and was approved by the University of Arizona IACUC (protocol #: 16-175).

## Author Contributions

EB and EM designed and conducted the research, analyzed the data, and wrote the paper. KL and BK helped with data collection, curation and supervision, and commented on drafts. JS and DD created the data collection tools, facilitated data collection and curation, and commented on drafts. All authors gave final approval for publication.

### Conflict of Interest Statement

The authors declare that the research was conducted in the absence of any commercial or financial relationships that could be construed as a potential conflict of interest.

## References

[1] Duffy DL Serpell JA Predictive validity of a method for evaluating temperament in young guide and service dogs. Appl Anim Behav Sci. (2012) 138:99–109. 10.1016/j.applanim.2012.02.011

[2] Goddard M Beilharz R Genetics of traits which determine the suitability of dogs as guide-dogs for the blind. Appl Anim Ethol. (1983) 9:299–315. 10.1016/0304-3762(83)90010-X

[3] Wiener P Haskell MJ Use of questionnaire-based data to assess dog personality. J Vet Behav. (2016) 16:81–5. 10.1016/j.jveb.2016.10.007

[4] Batt LS Batt M Baguley J McGreevy P Relationships between puppy management practices and reported measures of success in guide dog training. J Vet Behav. (2010) 5:240–6. 10.1016/j.jveb.2010.02.004

[5] Tomkins LM Thomson PC McGreevy PD Behavioral and physiological predictors of guide dog success. J Vet Behav. (2011) 6:178–87. 10.1016/j.jveb.2010.12.002

[6] Bray EE Sammel MD Cheney DL Serpell JA Seyfarth RM. Characterizing early maternal style in a population of guide dogs. Front Psychol. (2017) 8:175. 10.3389/fpsyg.2017.00175 28239365 PMC5301023

[7] Bray EE Sammel MD Cheney DL Serpell JA Seyfarth RM. The effects of maternal investment, temperament, and cognition on guide dog success. P Natl Acad Sci USA. (2017) 114:9128–33. 10.1073/pnas.1704303114 28784785 PMC5576795

[8] Goddard M Beilharz R Early prediction of adult behaviour in potential guide dogs. Appl Anim Behav Sci. (1986) 15:247–60. 10.1016/0168-1591(86)90095-X

[9] Svartberg K Shyness–boldness predicts performance in working dogs. Appl Anim Behav Sci. (2002) 79:157–74. 10.1016/S0168-1591(02)00120-X

[10] MacLean EL Hare B. Enhanced selection of assistance and explosive detection dogs using cognitive measures. Front Vet Sci. (2018) 5:1–14. 10.3389/fvets.2018.00236 30338264 PMC6180148

[11] MacLean EL Herrmann E Suchindran S Hare B Individual differences in cooperative communicative skills are more similar between dogs and humans than chimpanzees. Anim Behav. (2017) 126:41–51. 10.1016/j.anbehav.2017.01.005

[12] Bray EE Sammel MD Seyfarth RM Serpell JA Cheney DL Temperament and cognition in a population of adolescent guide dogs. Anim Cogn. (2017) 20:923–39. 10.1007/s10071-017-1112-8 28695349

[13] Berns GS Brooks AM Spivak M Levy K. Functional MRI in awake dogs predicts suitability for assistance work. Sci Rep. (2016) 7:43704. 10.1101/080325 28266550 PMC5339790

[14] Serpell JA Hsu Y. Development and validation of a novel method for evaluating behavior and temperament in guide dogs. Appl Anim Behav Sci. (2001) 72:347–64. 10.1016/S0168-1591(00)00210-0 11348683

[15] Hsu Y Serpell JA. Development and validation of a questionnaire for measuring behavior and temperament traits in pet dogs. J Am Vet Med Assoc. (2003) 223:1293–300. 10.2460/javma.2003.223.1293 14621216

[16] Duffy DL Serpell JA Behavioral assessment of guide and service dogs. J Vet Behav. (2008) 3:186–8. 10.1016/j.jveb.2007.12.010

[17] Wilsson E Sundgren P-E The use of a behaviour test for the selection of dogs for service and breeding, I: method of testing and evaluating test results in the adult dog, demands on different kinds of service dogs, sex and breed differences. Appl Anim Behav Sci. (1997) 53:279–95. 10.1016/S0168-1591(96)01174-4

[18] Harvey ND Craigon PJ Sommerville R McMillan C Green M England GC Test-retest reliability and predictive validity of a juvenile guide dog behavior test. J Vet Behav. (2016) 11:65–76. 10.1016/j.jveb.2015.09.005

[19] Arata S Momozawa Y Takeuchi Y Mori Y. Important behavioral traits for predicting guide dog qualification. J Vet Med Sci. (2010) 72:539–45. 10.1292/jvms.09-0512 20009419

[20] Harvey ND Craigon PJ Blythe SA England GC Asher L. An evidence-based decision assistance model for predicting training outcome in juvenile guide dogs. PLoS ONE (2017) 12:e0174261. 10.1371/journal.pone.0174261 28614347 PMC5470660

[21] Batt LS Batt MS Baguley JA McGreevy PD Factors associated with success in guide dog training. J Vet Behav. (2008) 3:143–51. 10.1016/j.jveb.2008.04.003

[22] Asher L Blythe S Roberts R Toothill L Craigon PJ Evans KM A standardized behavior test for potential guide dog puppies: methods and association with subsequent success in guide dog training. J Vet Behav. (2013) 8:431–8. 10.1016/j.jveb.2013.08.004

[23] Stekhoven DJ Bühlmann P. MissForest—non-parametric missing value imputation for mixed-type data. Bioinformatics (2012) 28:112–8. 10.1093/bioinformatics/btr597 22039212

[24] Shah AD Bartlett JW Carpenter J Nicholas O Hemingway H. Comparison of random forest and parametric imputation models for imputing missing data using MICE: a CALIBER study. Am J Epidemiol. (2014) 179:764–74. 10.1093/aje/kwt312 24589914 PMC3939843

[25] Moons KG Donders RA Stijnen T Harrell Jr FE. Using the outcome for imputation of missing predictor values was preferred. J Clin Epidemiol. (2006) 59:1092–101. 10.1016/j.jclinepi.2006.01.009 16980150

[26] Kuhn M Johnson K Applied Predictive Modeling (Vol. 26). New York, NY: Springer (2013). 10.1007/978-1-4614-6849-3

[27] Kuhn M Wing J Weston S Williams A Keefer C Engelhardt A caret: Classification and Regression Training. R package version 6.0–21. CRAN, Vienna, Austria (2015).

[28] Serpell JA Duffy DL. Aspects of juvenile and adolescent environment predict aggression and fear in 12-month-old guide dogs. Front Vet Sci. (2016) 3:49. 10.3389/fvets.2016.00049 27446937 PMC4916180

[29] Duffy DL Hsu Y Serpell JA Breed differences in canine aggression. Appl Anim Behav Sci. (2008) 114:441–60. 10.1016/j.applanim.2008.04.006

[30] MacLean EL Gesquiere LR Gruen ME Sherman BL Martin WL Carter CS. Endogenous oxytocin, vasopressin, and aggression in domestic dogs. Front Psychol. (2017) 8:1–14. 10.3389/fpsyg.2017.01613 29021768 PMC5624304

[31] Ilska J Haskell MJ Blott SC Sánchez-Molano E Polgar Z Lofgren SE. Genetic characterization of dog personality traits. Genetics (2017) 206:1101–11. 10.1534/genetics.116.192674 28396505 PMC5487251

[32] Liinamo AE van den Berg L Leegwater PA Schilder MB van Arendonk JA van Oost BA Genetic variation in aggression-related traits in Golden Retriever dogs. Appl Anim Behav Sci. (2007) 104:95–106. 10.1016/j.applanim.2006.04.025

[33] MacLean EL Snyder-Mackler N VonHoldt BM Serpell J Highly heritable and functionally relevant breed differences in dog behavior. BioRxiv [preprint] (2019). 10.1101/509315 PMC6790757 31575369

[34] Ruefenacht S Gebhardt-Henrich S Miyake T Gaillard C A behaviour test on German Shepherd dogs: heritability of seven different traits. Appl Anim Behav Sci. (2002) 79:113–32. 10.1016/S0168-1591(02)00134-X

[35] Wilsson E Sundgren P-E The use of a behaviour test for selection of dogs for service and breeding. II Heritability for tested parameters and effect of selection based on service dog characteristics. Appl Anim Behav Sci. (1997) 54:235–41. 10.1016/S0168-1591(96)01175-6

