# Supplementary material for: Predictive Models of Assistance Dog Training Outcomes Using the Canine Behavioral Assessment and Research Questionnaire and a Standardized Temperament Evaluation
Source: Front Vet Sci. 2019 Feb 27;6:49. doi: 10.3389/fvets.2019.00049 (PMC6400848; doi:10.3389/fvets.2019.00049)
Supplement: Supplementary file 1 [file Data_Sheet_1.PDF]

Appendix A

C-BARQ[101] scoring method

The C-*BARQ* provides a set of quantitative scores for the following fourteen different subscales or categories of behavior:

1. ***Stranger-directed aggression*:** Dog shows threatening or aggressive responses to strangers approaching or invading the dog's or owner's personal space, territory, or home range.
2. ***Owner-directed aggression*:** Dog shows threatening or aggressive responses to the owner or other members of the household when challenged, manhandled, stared at, stepped over, or when approached while in possession of food or objects.
3. ***Dog-directed aggression:*** Dog shows threatening or aggressive responses when approached directly by unfamiliar dogs.
4. ***Stranger-directed fear*:** Dog shows fearful or wary responses when approached directly by strangers.
5. ***Nonsocial fear*:** Dog shows fearful or wary responses to sudden or loud noises, traffic, and unfamiliar objects and situations.
6. ***Dog-directed fear*:** Dog shows fearful or wary responses when approached directly by unfamiliar dogs.
7. ***Separation-related behavior*:** Dog vocalizes and/or is destructive when separated from the owner, often accompanied or preceded by behavioral and autonomic signs of anxiety including restlessness, loss of appetite, trembling, and excessive salivation.
8. ***Attachment and attention-seeking*:** Dog maintains close proximity to the owner or other members of the household, solicits affection or attention, and displays agitation when the owner gives attention to third parties.
9. ***Trainability*:** Dog shows willingness to attend to the owner, obeys simple commands, learns quickly, fetches objects, responds positively to correction, and ignores distracting stimuli.
10. ***Chasing*:** Dog chases cats, birds, and/or other small animals, given the opportunity.
11. ***Excitability*:** Dog displays strong reaction to potentially exciting or arousing events, such as going for walks or car trips, doorbells, arrival of visitors, and the owner arriving home; has difficulty settling down after such events.
12. ***Touch sensitivity*:** Dog shows fearful or wary responses to potentially painful procedures, including bathing, grooming, nail-clipping, and veterinary examinations.
13. ***Energy level*:** Dog is energetic, "always on the go", and/or playful.
14. ***Dog rivalry*:** Dog shows aggressive or threatening responses to other familiar dogs in the household.

In addition, the C-BARQ provides useful information on the occurrence of a further 22 miscellaneous behavior problems ranging from coprophagia to stereotypic spinning/tail-chasing.

Each subscale is represented by a number of 5-point scales (questions). Some are graduated scales that measure severity of particular behaviors (e.g. aggression) and are numbered from 0–4 in the questionnaire. The remainder are frequency scales which should be scored as: Never = 0, Seldom = 1, Sometimes = 2, Usually = 3 and Always = 4, **except for scales 5, 6 and 7 in Section 1. FOR THESE SCALES ONLY, reverse the scores to: Never = 4, Seldom = 3, etc.**

To calculate behavior subscale scores, use the following formulae:

“Excitability” score = (items 63 + 64 + 65 + 66 + 67 + 68)/6

“Nonsocial fear” score = (items 38 + 41 + 42 + 44 + 47 + 48)/6

“Trainability” score = (items 1 + 2 + 3 + 4 + 5 + 6 + 7 + 8)/8—remember to reverse scoring order for items 5, 6 & 7 (see above).

“Separation-related problems” score = (items 55 + 56 + 57 + 58 + 59 + 60 + 61 + 62)/8

Items 1–77 & 92–93 cannot be removed from the questionnaire without potentially reducing the reliability and/or validity of one or other of the behavior subscales. Other “Miscellaneous” items are optional, and can be removed from the questionnaire as desired. If retained, they should be scored individually, 0–4.

NB: This version of the C-*BARQ* has been modified since Hsu & Serpell (2003) to improve the reliability of some existing factors, and to include new “Dog rivalry (familiar dog aggression)” and “Energy” factors. The subscales “Dog rivalry”, “Chasing”, “Touch sensitivity”, “Trainability”, “Energy” and “Excitability” have not been formally validated, although they have been shown to have predictive validity in long-term studies of guide dogs (Duffy & Serpell, 2008).

© James A. Serpell
