# Supplementary material for: Predictive Models of Assistance Dog Training Outcomes Using the Canine Behavioral Assessment and Research Questionnaire and a Standardized Temperament Evaluation
Source: Front Vet Sci. 2019 Feb 27;6:49. doi: 10.3389/fvets.2019.00049 (PMC6400848; doi:10.3389/fvets.2019.00049)
Supplement: Supplementary file 2 [file Data_Sheet_2.PDF]

Appendix B

Dog Name: ________________________________ CCI ID:_______________________

Evaluator: _________________________ Date: ____________________ **Region**:___________

Response to Physical Exam

Stranger calmly handles all parts of the dogs body as in a vet exam, giving positive support during handling. Final manipulation – rollover onto side.

EASE OF HANDLING (Check as appropriate)

- ○ No resistance, immediately relinquishes to handler (0)
- ○ Slight resistance initially, quickly relinquishes to handler (1)
- ○ Resists initially then relinquishes (2)
- ○ Resists throughout one or two components of the exam (e.g. face, body, rollover) (3)
- ○ Resists throughout entire exam (4)

GENERAL DEMEANOR

- ○ Calm (A)
- ○ Playful/excitable (B)
- ○ Active submissive/solicitous (C)
- ○ Passive submissive (D)
- ○ Fearful/anxious (E)
- ○ Defensive aggressive (F)

OTHER BEHAVIOR

Body tension/tone:	Vocalization:	Tail position:
○ Relaxed (0)	○ None (0)	○ Normal/relaxed (0)
○ Slightly tense (1)	○ Whining/yelping (1)	○ Somewhat tucked (1)
○ Tense (2)	○ Growling (2)	○ Tightly tucked (2)

Comments:

Response to Looming Object

Dog is walked towards black plastic garbage bag that falls off a 3 to 4 ft wall or ledge towards the dog. Handler does not initially cue dog to continue towards object.

INITIAL REACTION (Check as appropriate):

- ○ No reaction to object (0)
- ○ Acknowledges bag falling, but shows no startle reaction (0)
- ○ Startles & stops but does not retreat (1)
- ○ Startles and retreats a few steps (2)
- ○ Retreats to end of leash but keeps eye on object (3)
- ○ Bolts to end of leash without glancing back (4)

Handler allows dog freedom to approach or freedom to retreat to end of 6-foot leash.

RECOVERY PHASE:

- ○ Approaches immediately (0)
- ○ Approaches cautiously without encouragement (1)
- ○ Approaches reluctantly after encouragement (2)
- ○ Refuses to approach object, even with encouragement (3)

OTHER BEHAVIOR:

Barks or growls at object:	○ No (0)	○ Yes (1)
Increase in activity/excitability level:	○ No (0)	○ Yes (1)

Dog is walked past the stationary fallen bag one additional time

SECOND WALK BY:

- ○ Ignores or acknowledges object, and moves on (0)
- ○ Avoids object (1)

Comments:

Response to Sudden Noise

INITIAL REACTION (Check as appropriate):

- ○ Stops and orients to sound, no other reaction (0)
- ○ Stops and crouches or flinches, but does not move away (1)
- ○ Quick start away then stops, never looks away (2)
- ○ Retreats from noise up to 10 feet, looks away from sound(3)
- ○ Retreats more than 10 feet, looks away (4)

RECOVERY PHASE: *(Investigation)*

- ○ Advances to object independently (0)
- ○ Advances to object when handler is halfway to object (1)
- ○ Advances to object when handler is at object (2)
- ○ Advances to object after handler begins speaking (3)
- ○ Does not approach or waits until handler touches object (4)

Conclusion: (*Second Pass*)

- ○ No sign of reaction, change of pace, etc. (0)
- ○ Slight reaction or change of pace (1)
- ○ Moderate increase in pace or ears flattened while passing (2)
- ○ Tight leash, rushes past object (3)

Other:

Barks or growls at object: ○ No (0) ○ Yes (1)

Comments:

Response to 'Prey'

INITIAL REACTION (Check as appropriate):

- ○ No reaction to object, does not start (0)
- ○ Starts after object slowly (1)
- ○ Starts immediately (2)

RECOVERY PHASE: *(Speed of Chase)*

- ○ N/A (does not start) (0)
- ○ Slow chase that stops before completion (1)
- ○ Slow to moderate speed over entire course (2)
- ○ High speed over entire course (3)
- ○ High speed, catches object before third pole (4)

Conclusion: *(Conclusion of Chase)*

- ○ N/A (does not start) (0)
- ○ No interest/ does not reach rag (1)
- ○ Sniffs but does not pick up rag (2)
- ○ Picks up rag cautiously (3)
- ○ Picks rag right up, may drop it right away (4)
- ○ Picks rag right up, shakes, carries or continues to hold (5)

Other:

Barks or growls at object: ○ No (0) ○ Yes (1)

Comments:

Response to Unfamiliar Dog

Medium to large sized dog (dissimilar breed) becomes visible at distance of approx. 10 yards. Dog is walked towards distraction 3x, coming closer to it on each approach. (20, 10, 2 ft)

INITIAL REACTION (Check as appropriate):

- ○ No reaction to other dog (0)
- ○ Acknowledges other dog briefly, but shows little or no change in pace (0)
- ○ Shows interest and increased walking pace toward other dog (1)
- ○ Rushes or attempts to rush toward other dog (2)
- ○ Reluctant to approach dog, stops or slows, or falls behind handler (2)

Handler silently about turns using collar cues to bring dog away from distraction. Handler is silent prior to leash action, then may praise dog for responding.

RECALL (when asked to leave other dog):

- ○ Readily follows handler away from dog with minimal collar pressure (0)
- ○ Follows handler after one or two moderate collar actions (1)
- ○ Follows handler after numerous collar actions (2)
- ○ Requires numerous firm collar actions to follow handler (3)
- ○ Avoids approaching other dog (3)

OTHER BEHAVIOR:

Hackles? ○ No (0) ○ Yes (1)
Barks or growls at other dog: ○ No (0) ○ Yes (1)
Tail position: ○ Normal/relaxed (0) ○ Elevated (1) ○ Tucked (1)

Comments:

Response to Threatening Stranger

Dog is walked towards hooded, masked stranger dressed in hooded coat, carrying cane. Stranger is visible to dog at a distance of 10 yards. Stranger moves 10 feet towards dog while shouting (recorded voice) and waving stick at dog, in threatening manner.

INITIAL REACTION (Check as appropriate):

- ○ Little or no reaction to stranger, or approaches without hesitation (0)
- ○ Alerts to stranger and stops; does not retreat (0)
- ○ Startles and retreats a few steps (1)
- ○ Retreats to end of leash but keeps eye on stranger (2)
- ○ Bolts to end of leash without glancing back (3)

Following initial response, stranger becomes passive, faces away from dog, then kneels and engages in friendly interaction.

RECOVERY PHASE:

- ○ Approaches stranger immediately (0)
- ○ Approaches cautiously without encouragement (1)
- ○ Approaches reluctantly after encouragement (2)
- ○ Refuses to approach stranger, even with encouragement (3)

OTHER BEHAVIOR:

Hackles? ○ No (0) ○ Yes (1)
Barks or growls at stranger: ○ No (0) ○ Yes (1)
Increase in activity/excitability level: ○ No (0) ○ Yes (1)

Comments:

Save As

WEATHER DURING TEST:

General Conditions ____________________ Wind ________________

Temperature _____ Time of day _____________________
